# The Protective Effects of 18*β*-Glycyrrhetinic Acid on Imiquimod-Induced Psoriasis in Mice via Suppression of mTOR/STAT3 Signaling

**DOI:** 10.1155/2020/1980456

**Published:** 2020-08-27

**Authors:** Haiming Chen, Huazhen Liu, Bin Tang, Yuchao Chen, Ling Han, Jingjie Yu, Yuhong Yan, Chuanjian Lu

**Affiliations:** ^1^State Key laboratory of Dampness Syndrome of Chinese Medicine, The Second Affiliated Hospital of Guangzhou University of Chinese Medicine, Guangzhou 510115, China; ^2^Guangdong Provincial Key Laboratory of Clinical Research on Traditional Chinese Medicine Syndrome, Guangzhou 510115, China

## Abstract

Psoriasis is recognized as an autoimmune and inflammatory dermatosis, which is estimated to affect 2-3% of the population worldwide. 18*β*-Glycyrrhetinic acid (GA), one of the main ingredients of Licorice (Glycyrrhiza *glabra* L.), has been shown to have numerous pharmacological effects such as antioxidative, antitumor, and anti-inflammatory activities. However, it remains to be explored whether GA has antipsoriatic effect on psoriasis. In this study, we evaluated the protective effect of GA on psoriasis and its mechanisms of action in imiquimod-induced psoriasis-like mouse model. Results indicated that GA dramatically improved psoriatic lesions and reduced psoriasis area and severity index scores. GA also suppressed the mRNA levels of IL-6, TNF-*α*, IL-17, IL-23, and IL-1*β* in the skin and increased the proportion of CD4+ Foxp3+ regulatory T cells (Tregs) in both lymph nodes and spleens. Its anti-inflammatory and immunomodulatory activities may be related to its suppression of the STAT3 and mTOR signaling. In conclusion, GA ameliorated the symptoms of psoriasis, at least in part, through inhibition of inflammatory cytokines and STAT3/mTOR signaling and activation of Tregs in both lymph nodes and spleens. These effects are expected to be beneficial in the treatment and prevention of psoriasis.

## 1. Introduction

Psoriasis, which is deemed as a chronic inflammatory immune-related skin disease, has a prevalence rate of 2-3% in the world [1, 2]. The major pathophysiology of psoriasis is characterized by hyperkeratosis of the epidermis, immune cell infiltration in the dermis and epidermis, and angiogenesis in the dermis [3]. Meanwhile, it has been proved that psoriatic patients have a higher incidence of arthritis, cardiovascular disease, metabolic disorders, and even cancer, which indicates psoriasis may be a systemic disorder rather than only a skin disorder [4–6]. A variety of factors have been demonstrated to be associated with psoriasis, including genetic factors, infection factors, and environmental factors. Besides, obesity and weight gain have been shown to be important risk factors for the incidence of psoriasis and the trigger of severity [7].

For the treatment strategies for psoriasis, some topical therapies are used in mild psoriasis, and phototherapy or systemic agents are often used for severe patients [8]. Nowadays, biologic therapies have also been developed and approved for the treatment of psoriasis [9]. However, these synthetic and biological agents are of high cost and might possess unwanted side effects [9, 10]. Therefore, the safety, economy, and effectiveness have become the most central concerns for the long-term treatment for psoriasis.

During recent decades, Chinese herbal medicine, which has a long history and has been reported to be effective and safe for psoriasis [11], has been used as popular strategies for treating psoriatic patients. Licorice (Glycyrrhiza *glabra* L.), a perennial herb widely grown in China, Japan, and Russia, is one of the most widely used herbal medicines in the world.

**Figure 1:**
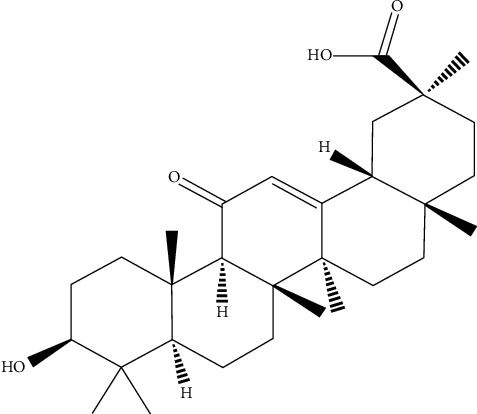
The chemical structure of GA.

18*β*-Glycyrrhetinic acid (GA, structure shown in Figure 1), an aglycone and active metabolite of glycyrrhizic acid, has been shown to have a series of pharmacological effects such as antioxidative [12, 13], antitumor [14–16], and anti-inflammatory [17, 18] activities. Moreover, in recent years, this compound has also been reported to show some protective effect in skin disorders [19–21]. However, no report has been issued on the protective effects of GA against imiquimod-induced psoriasis-like skin *in vivo*. Hence, the aim of this present study was to investigate the potential protective effects of GA on imiquimod-induced mice psoriasis and explored its underlying molecular mechanisms.

## 2. Materials and Methods

### 2.1. Animals

Male BALB/c mice (6–8 weeks old, weighing 20 ± 2 g) were purchased from the Center of Laboratory Animals of Southern Medical University (Guangzhou, China). Mice were housed in a standard housing room under controlled conditions (maintained at 22 ± 2°C, 45 to 55% relative humidity) and provided free access to food and water under a specific pathogen-free (SPF) environment. The animal protocols were approved by the Animal Experimental Ethics Committee of Guangdong Provincial Hospital of Chinese Medicine.

### 2.2. Chemical and Reagents

Methotrexate (MTX) was purchased from Shanghai Xinyi Pharmaceutical Factory (Shanghai, China). Imiquimod cream was obtained from Sichuan Mingxin Pharmaceutical Co., LTD (Sichuan, China). 18*β*-Glycyrrhetinic acid was bought from Sigma-Aldrich (St. Louis, MO, USA). Enhanced chemiluminescence (ECL) reagent was purchased from Millipore (Billerica, MA, USA).

### 2.3. Administration of Drugs

BALB/c mice were randomly divided into 5 groups (*n* = 6). The groups included the control, vehicle, MTX (1 mg/kg/day), and GA (60 and 120 mg/kg/day, respectively). The control group was normal mice that were totally untreated. GA and MTX were dissolved in distilled water and administered to mice in the GA or MTX group, respectively, while distilled water was orally given to control and vehicle groups. Vehicle and treatment groups were given topical administration of imiquimod cream to induce psoriasis. The topical treatment and oral administration were applied for 7 consecutive days.

### 2.4. Imiquimod-Induced Psoriasis-Like Mouse Model

According to our previous study [22], mice were topically administrated with a dose of 62.5 mg of 5% imiquimod cream which was applied to a shaved area (3 cm × 2.5 cm) on their back for 7 consecutive days.

The Psoriasis Area and Severity Index (PASI) concerning skin erythema, scaling, and thickness was used to monitor the grade of the psoriasis-like lesion severity. Parameters were scored independently on a scale ranging from 0 to 4 according to the clinical signs, in which “0” represents none, “1” denotes slight, “2” means moderate, “3” depicts marked, and “4” indicates very marked clinically.

### 2.5. Histological Analysis and Immunohistochemistry

The dorsal skin, liver, and kidney samples of the mice were fixed in 4% paraformaldehyde and embedded in paraffin. Sections (5 *μ*m) were then made and stained with hematoxylin and eosin (H&E) for histological analysis. For immunohistochemical staining, antigen retrieval was conducted with citrate buffer (pH 6.0) followed by treatment with 3.0% H_2_O_2_ to quench endogenous peroxidase activity. The sections were incubated overnight at 4°C with specific primary antibodies against Ki67 and CD3. The sections were then incubated with biotinylated secondary antibodies for 1 h at room temperature followed by diaminobenzidine staining and hematoxylin counterstaining.

### 2.6. Measurements of TNF-*α*, IL-6, IL-17, IL-23, and IL-1*β* via RT-PCR

The skin mRNA levels of TNF-*α*, IL-6, IL-17, IL-23, and IL-1*β* were evaluated by RT-PCR. Total mRNA was isolated from mouse skin tissue using Trizol reagents, and mRNA was then subjected to reverse transcription to cDNA. The primer sequences were shown in Table 1. The relative mRNA expression levels of cytokines versus GAPDH were measured using an ABI 7500 Fast Real-Time PCR System (Thermo Fisher Scientific, USA).

### 2.7. Western Blotting Analysis

Total protein from mouse skin samples were acquired with RIPA lysis buffer followed by centrifugation (12,000 rpm and 15 min) at 4°C. Equal amounts of protein from each treatment group were subjected to fractionation by SDS-PAGE and electro-transferred to PVD membranes. The membranes were then blocked with 5% (*w*/*v*) skim milk in TBS-T containing 0.1% Tween-20 at room temperature for 2 h and subsequently incubated with primary antibody at 4°C overnight. Then, the membranes were washed with TBS-T and blotted with the appropriate secondary antibody for 1 h. Finally, the protein bands were detected using the enhanced chemiluminescence (ECL). The band intensity was quantified using Image J software (NIH Image, Bethesda, MD, USA), and GAPDH was used as the loading control.

### 2.8. CD4+Foxp3+ Treg Quantification Using Flow Cytometry

Lymph node and spleen cells from mice were harvested after treatments. Cells were isolated and stained using surface markers (anti-CD4-PE, eBioscience) and then an intracellular marker (anti-Foxp3-APC, eBioscience) using intracellular fixation/permeabilization kit (eBioscience, San Diego, CA). And CD4+Foxp3 Tregs were analyzed using a FACSCalibur (BD, Biosciences).

### 2.9. Statistical Analysis

The data were statistically evaluated by one-way analysis of variance (ANOVA) followed by Dunnett's test and denoted as means ± standard deviation (SD). Statistically significant differences were identified as either *P* < 0.05 or *P* < 0.01. All analyses were carried out by GraphPad Prism 5.0 (GraphPad Software, La Jolla, CA, USA).

## 3. Results

### 3.1. GA Ameliorates Imiquimod-Induced Psoriatic Skin Lesion in Mice

**Figure 2:**
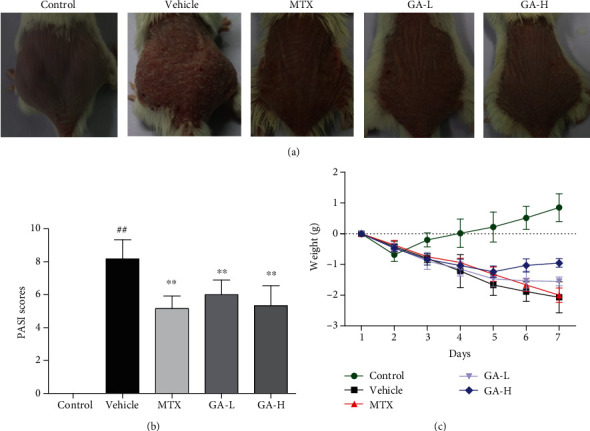
GA reduces the PASI score and ameliorates the skin lesion in IMQ-induced psoriasis-like mice. (a) The representatives of photos of dorsal skin in IMQ-induced psoriasis-like mice 7 days after IMQ-treatment without or with GA. (b) The PASI scores of the skin lesion in IMQ-induced psoriasis-like mice after treatment. (c) The daily mouse weight during the treatment. Data are presented as the means ± SD (*n* = 6, ^#^*P* < 0.05 and ^##^*P* < 0.01 vs. control group, ^∗^*P* < 0.05 and ^∗∗^*P* < 0.01 vs. vehicle group). (GA-L: low-dose of GA; GA-H: high-dose of GA).

In order to evaluate the antipsoriatic effects of GA, imiquimod-induced psoriasis-like mouse models were applied. The morphological observations and PASI scores were shown in Figure 2. The control group devoid of imiquimod had normal skin without any sign of inflammation, while the vehicle group showed psoriasis-like lesions, including skin erythema, scaling, and thickening. Overall skin lesions of mice and average PASI scores were significantly reduced after treatment with GA or MTX compared with the vehicle group.

### 3.2. Histological Evaluations

**Figure 3:**
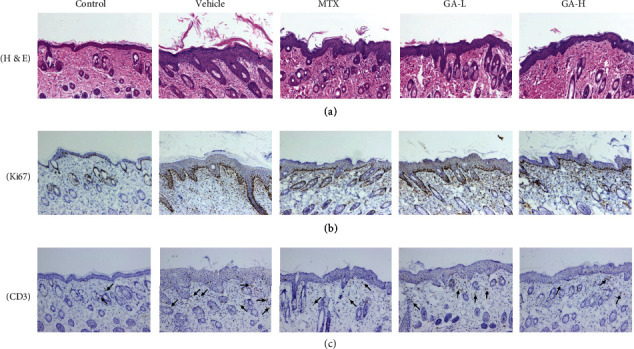
Histological analysis and immunohistochemistry assay. Different treatments (MTX and GA) were applied to the mice for 7 days. H&E staining of the dorsal skin lesion (a) in different treatment groups. Immunohistochemical images of Ki67 (b) or CD3 (c) staining (magnification: 100x) of dorsal skin in control or psoriatic mice 7 days after treatment.

Histological examinations *via* H&E staining were performed on the lesion skin 7 days after treatment with GA. As shown in Figure 3(a), mice skin tissue slide in the control group showed normal smooth epidermis without any inflammation or lesion. While significant pathological changes characterized by increased acanthosis, hyperkeratosis of the epidermis, and abundant inflammatory infiltrates were found in the mice skin of the vehicle group. However, treatment with either MTX or GA resulted in much smoother epidermis with less parakeratosis and reduced epidermal thickening as compared with the vehicle group.

### 3.3. Effects of GA on Immunohistochemistry of Ki67 and CD3

Hyperproliferation and inflammation infiltration to the skin is crucial to the pathogenesis of psoriasis; therefore, we detected the protein expression of Ki67 and CD3 in the skin. It was observed from the results (Figures 3(b) and 3(c)) that Ki67 and CD3 expression in the vehicle group was significantly higher than those in the control group. In contrast, the expression of Ki67 and CD3 was obviously decreased in mice treated with either low-dose or high-dose of GA.

### 3.4. GA Suppresses mRNA Expression of pro-Inflammatory Cytokines in Imiquimod-Treated Psoriatic Mice

**Figure 4:**
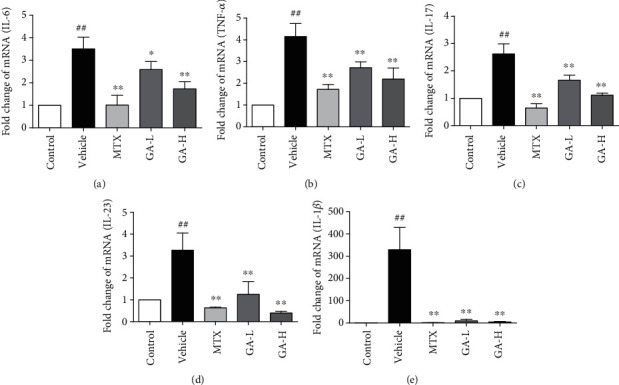
Effect of GA on the mRNA expression of inflammatory cytokines in imiquimod-induced psoriasis. Mice were administrated with distilled water, MTX and GA (60 and 120 mg/kg, respectively) for 7 days, and topically administrated with imiquimod. The mRNA levels of IL-6 (a), TNF-*α* (b), IL-17 (c), IL-23 (d), and IL-1*β* (e) in the skin were determined using RT-PCR. Data shown are the means ± SD (*n* = 3, ^#^*P* < 0.05 and ^##^*P* < 0.01 compared with control group, ^∗^*P* < 0.05 and ^∗∗^*P* < 0.01 compared with vehicle group).

To analyze the potential anti-inflammatory effect of GA, the mRNA expression of TNF-*α*, IL-6, IL-17, IL-23, and IL-1*β* in skin tissue was determined using RT-PCR. As shown in Figure 4, the mRNA expression of TNF-*α*, IL-6, IL-17, IL-23, and IL-1*β* after treatment with imiquimod was significantly enhanced as compared with those in other groups. While the mRNA levels of these cytokines after administration of GA were obviously lower as compared with the imiquimod-treated group without GA (vehicle). Moreover, the mRNA expression of TNF-*α*, IL-6, IL-17, and IL-1*β* treated with MTX was lower than that of GA-L or GA-H groups, and IL-23 level of MTX group was lower than that of GA-L but higher than that of the GA-H group.

### 3.5. GA Increases CD4+Foxp3+ Tregs in Psoriatic Mice

**Figure 5:**
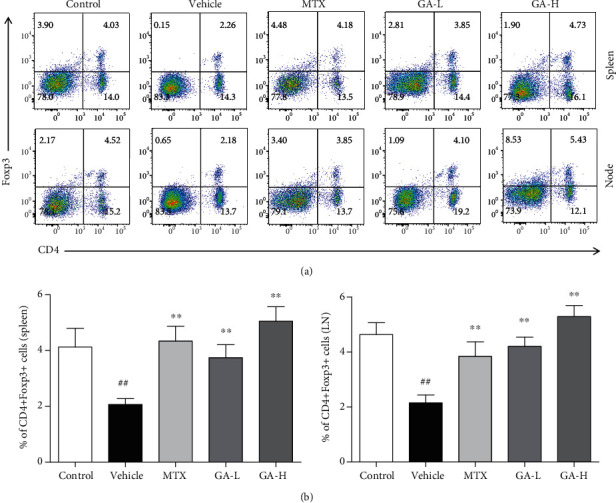
GA induces CD4+Foxp3+ Tregs in imiquimod-induced psoriasis-like mice. Effects of GA on CD4+Foxp3+ Treg frequency in spleens and lymph nodes of IMQ-induced psoriasis-like mice were observed. Spleen and lymph node cells were isolated from imiquimod-induced psoriasis-like mice 7 days after treatment with GA or MTX. To quantify CD4+Foxp3+ Tregs, cells were stained for CD4 surface and intracellular Foxp3 makers. Data shown are the means ± SD (*n* = 4, ^#^*P* < 0.05 and ^##^*P* < 0.01 compared with control group, ^∗^*P* < 0.05 and ^∗∗^*P* < 0.01 compared with vehicle group).

CD4+Foxp3+ Tregs which serve as a small subset of T cells play an important role in maintaining immunological tolerance and preventing autoimmune diseases in psoriasis. Therefore, we determined the effect of GA on the frequency of Tregs in lymph nodes and spleens of psoriasis-like mice using FACS analyses. As displayed in Figure 5, the frequency of CD4+Foxp3+ Tregs in both lymph nodes and spleens increased significantly after treatment with either low or high doses of GA or MTX compared with that of the vehicle group.

### 3.6. GA Inhibits mTOR/STAT3 Signaling in the Skin with Psoriasis-Like Lesions

**Figure 6:**
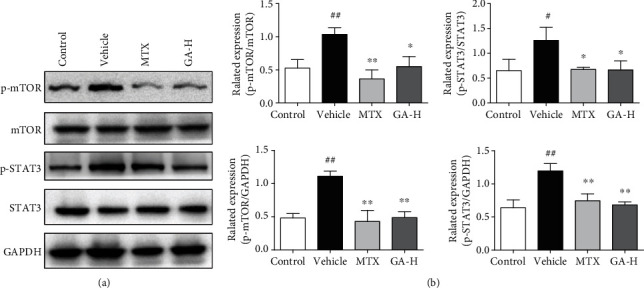
GA inhibits p-mTOR and p-STAT3 protein expression in the skin of IMQ-induced psoriasis-like mice. Impacts of GA on the protein expression of p-mTOR and p-STAT3 in skin tissue of IMQ-induced psoriasis-like mice were determined using Western blotting after GA treatment. The expression of p-mTOR, mTOR, p-STAT3, and STAT3 was detected using Western blotting (a). The densitometry analyses of the immunoblotting are shown as p-mTOR/mTOR, p-mTOR/GAPDH, p-STAT3/STAT3, and p-STAT3/GAPDH (b). Data shown are the means ± SD (*n* = 3, ^#^*P* < 0.05 and ^##^*P* < 0.01 vs. control group, ^∗^*P* < 0.05 and ^∗∗^*P* < 0.01 vs. vehicle group).

Since GA suppressed proinflammatory cytokine expression and elevated the frequency of CD4+Foxp3+ Tregs, we further explored the mechanisms underlying its antipsoriatic or anti-inflammatory effects. STAT3 signaling is a prominent therapeutic target for treating inflammatory diseases. mTOR signaling is known to be important in regulating the innate and adaptive immune responses and the process of Treg cell differentiation. Thus, Western blotting analysis was performed to evaluate the effect of GA on STAT3/mTOR signaling pathway involving protein expression of p-STAT3 and p-mTOR. As shown in Figure 6, the expression of p-STAT3 and p-mTOR markedly increased after treatment with imiquimod compared to that of the control group. However, treatment with GA markedly suppressed the expression of p-STAT3 and p-mTOR (*P* < 0.05 and *P* < 0.01, respectively) compared to the vehicle group, and the relative levels of p-STAT3/STAT3 and p-mTOR/mTOR of the MTX group were lower than those of GA group.

## 4. Discussion

Although the cause of psoriasis is incompletely understood, its most important pathological features include epidermal keratinocyte hyperproliferation, immune cell infiltration, and angiogenesis in hyperplastic dermal [23]. In our study, we demonstrated that the protective effect of GA in imiquimod-induced psoriasis-like mice and revealed its possible mechanisms of action.

It has been reported that repeated application of imiquimod, an agonist for toll-like receptors 7/8, on mice skin could cause obvious inflammation with leukocyte incursion and severe epidermal hyperplasia, closely resembling human psoriasis-like pathologic changes [24–26]. Therefore, we observed the therapeutic effect of GA on psoriasis-like lesions in mice.

In this study, we established the model of imiquimod-induced psoriasis and observed that GA significantly lowered the PASI scores, decreased the epidermal hyperplasia and epidermal thickening compared to the vehicle group, indicating that GA effectively ameliorated imiquimod-induced murine psoriasis. Meanwhile, it was evident that neither liver nor kidney toxicity (Figure S1) to mice was observed after oral administration of GA for 7 consecutive days.

Many proinflammatory cytokines and chemokines such as TNF-*α*, IL-6, and IL-1*β* play a cardinal role in the aggravation of psoriatic lesions [27]. Topical application of imiquimod on the mice not only induces skin inflammation but also generates overproduction of inflammatory mediators. It has been proved that IL-23/IL-17 axis plays a central role in the development of psoriasis [28, 29], and excessive amounts of inflammatory cytokines IL-17 and IL-23 have been found in human psoriasis and imiquimod-induced murine psoriasis [25, 30]. In our study, it was found that imiquimod could upregulate the expression of inflammatory cytokines and GA could significantly inhibit the mRNA levels of TNF-*α*, IL-6, IL-17, IL-23, and IL-1*β*.

It has been proved that STAT3 signaling pathway, which is associated with inflammatory diseases, is required for proinflammatory cytokine production and keratinocyte proliferation within psoriatic skin lesions [31]. Activation of STAT3 plays a critical role in psoriasis. Studies have shown that STAT3 is continuously highly activated in psoriasis compared to other nonpsoriatic, inflammatory skin disorders displaying epidermal hyperplasia. In addition, transgenic mice, in which keratinocytes express a constitutively active form of STAT3, develop psoriasis-like skin lesions spontaneously. In our study, we found that GA could suppress STAT3 phosphorylation, which suggested that the anti-inflammatory effect of GA might be associated with inhibition of the STAT3 pathway.

It has been shown that mTOR signaling plays a critical role in psoriasis pathogenesis [32, 33]. Activation of mTOR signaling in psoriatic skin, particularly in keratinocytes, has been reported previously [34]. mTOR inhibitors have been tested in the animal models and clinics for the treatment of psoriasis [33, 34]. Preliminary data have suggested that blocking mTOR signaling reduces disease severity [33]. Previous studies have shown that the PI3K/AKT/mTOR pathway is negatively correlated with the differentiation of iTreg cells. When AKT is continuously activated, the expression of FoxP3 is significantly downregulated, which in turn inhibits the differentiation of Treg cells. However, PI3K or AKT inhibitors for PI3K/AKT/mTOR signaling can enhance iTreg cell differentiation [35, 36]. In our study, it was shown that GA inhibited the phosphorylation of mTOR and increased the CD4+FoxP3+ Treg frequency in spleen and lymphonodus in mice with imiquimod-induced psoriasis. It was hypothesized that GA induced CD4+Foxp3+ Tregs possibly via inhibiting the mTOR signaling.

In conclusion, we found that treatment with GA effectively alleviated psoriasis-like mice skin lesion induced by topical administration of imiquimod. GA reduced the mRNA expression of major proinflammatory cytokines in the psoriatic mouse skin. Moreover, the therapeutic effect of GA was intimately associated with a significant increase in CD4+FoxP3+ Treg frequency in the spleen and lymph nodes and suppression of the STAT3/mTOR signaling.

## Figures and Tables

**Table 1:** Primer sequences of target genes.

Target gene	Primer sequence (5′→3′)
TNF-*α* (forward)	ACTGATGAGAGGGAGGCCAT
TNF-*α* (reverse)	CCGTGGGTTGGACAGATGAA
IL-6 (forward)	TTCTTGGGACTGATGCTGGT
IL-6 (reverse)	CCTCCGACTTGTGAAGTGGT
IL-17 (forward)	TCAAAGCTCAGCGTGTCCAA
IL-17 (reverse)	TCTTCATTGCGGTGGAGAGTC
IL-23 (forward)	CAAAGGATCCGCCAAGGTCT
IL-23 (reverse)	GGAGGTGTGAAGTTGCTCCA
IL-1*β* (forward)	TGCCACCTTTTGACAGTGATG
IL-1*β* (reverse)	AAGGTCCACGGGAAAGACAC
GAPDH (forward)	CAGGTTGTCTCCTGCGACTT
GAPDH (reverse)	TATGGGGGTCTGGGATGGAA

## Data Availability

The data used to support the findings of this study are available from the corresponding author upon request.

## Abbreviations

GA: 18*β*-Glycyrrhetinic acid
MTX: Methotrexate
Treg: Regulatory T cell

## Supplementary Materials

Supplementary Materials Figure S1: histological analysis of liver and kidney after administration with GA.
