# Supplementary figures and images for: The Protective Effects of 18β-Glycyrrhetinic Acid on Imiquimod-Induced Psoriasis in Mice via Suppression of mTOR/STAT3 Signaling
Source: J Immunol Res. 2020 Aug 27;2020:1980456. doi: 10.1155/2020/1980456 (PMC7474397; doi:10.1155/2020/1980456)

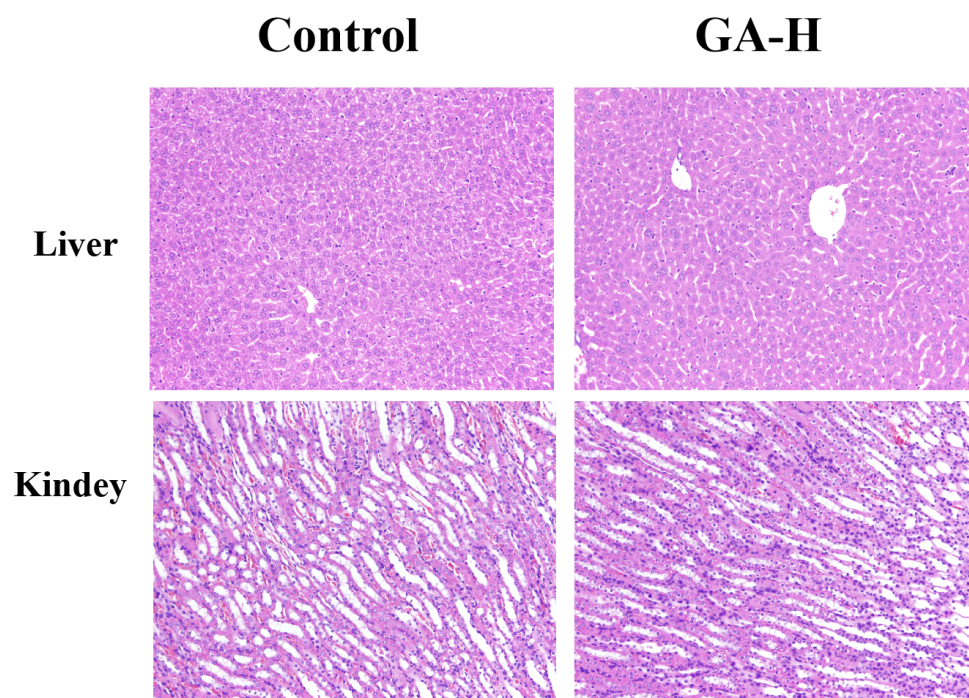

**Figure S1. Histological analysis of liver and kidney after administration with GA .**

Supplement: Supplementary Materials — Figure S1: histological analysis of liver and kidney after administration with GA. [file 1980456.f1.pdf]
